# Unveiling the Microbiome Landscape: A Metagenomic Study of Bacterial Diversity, Antibiotic Resistance, and Virulence Factors in the Sediments of the River Ganga, India

**DOI:** 10.3390/antibiotics12121735

**Published:** 2023-12-14

**Authors:** Ajaya Kumar Rout, Partha Sarathi Tripathy, Sangita Dixit, Dibyajyoti Uttameswar Behera, Bhaskar Behera, Basanta Kumar Das, Bijay Kumar Behera

**Affiliations:** 1 Aquatic Environmental Biotechnology and Nanotechnology Division, ICAR—Central Inland Fisheries Research Institute, Kolkata 700120, WB, India; ajayabi2012@gmail.com (A.K.R.); basantakumar@gmail.com (B.K.D.); 2 Department of Biosciences and Biotechnology, Fakir Mohan University, Balasore 756089, OD, India; drbhaskarbehera@gmail.com; 3 Faculty of Biosciences and Aquaculture, Nord University, Universitetsalléen 11, 8026 Bodø, Norway; rabul92@gmail.com; 4 Center for Biotechnology, School of Pharmaceutical Sciences, Siksha ‘O’ Anusandhan (Deemed to Be University), Bhubaneswar 751030, OD, India; sangitadixit2011@gmail.com (S.D.); dibya01bioinfo@gmail.com (D.U.B.)

**Keywords:** metagenomics, diversity, ganga, sediment, AMR, virulence factors

## Abstract

The global rise in antibiotic resistance, fueled by indiscriminate antibiotic usage in medicine, aquaculture, agriculture, and the food industry, presents a significant public health challenge. Urban wastewater and sewage treatment plants have become key sources of antibiotic resistance proliferation. The present study focuses on the river Ganges in India, which is heavily impacted by human activities and serves as a potential hotspot for the spread of antibiotic resistance. We conducted a metagenomic analysis of sediment samples from six distinct locations along the river to assess the prevalence and diversity of antibiotic resistance genes (ARGs) within the microbial ecosystem. The metagenomic analysis revealed the predominance of Proteobacteria across regions of the river Ganges. The antimicrobial resistance (AMR) genes and virulence factors were determined by various databases. In addition to this, KEGG and COG analysis revealed important pathways related to AMR. The outcomes highlight noticeable regional differences in the prevalence of AMR genes. The findings suggest that enhancing health and sanitation infrastructure could play a crucial role in mitigating the global impact of AMR. This research contributes vital insights into the environmental aspects of antibiotic resistance, highlighting the importance of targeted public health interventions in the fight against AMR.

## 1. Introduction

The river Ganga, esteemed for its immense religious, cultural, spiritual, and ritual significance in India, is considered a sacred water body. It is exposed to considerable human impact as it serves nearly 400 million people, with a population density of around 520 individuals per square kilometer, as per recent estimates [1,2]. The river has increasingly become a focal point of pollution issues, notably the discovery of bacterial strains highly resistant to standard antibiotics [3,4]. Concurrently, research has identified bacterial and fungal strains within the Ganga’s sediments capable of bioremediating potential and beneficial microbiomes [5,6]. Factors like rapid urbanization, industrial activities, population surges, and the release of agricultural waste have intensified pollution in the river. Additionally, the practice of mass religious bathing, drawing large crowds, including the elderly, consuming medicinal products, further impacts the river’s condition. The Ganga’s banks also attract tourists for spiritual pursuits, water sports, and hiking activities. Human settlements and industrial establishments within the river basin markedly affect the water quality, influencing the river’s microbial composition [7,8,9,10,11]. Pollutants and toxic substances in the river significantly alter the dynamics of its microbial communities. A major concern is the rise of antibiotic resistance, a global health threat. Human activities have led to an increased prevalence of antibiotic resistance genes (ARGs) in the river [2]. Previous studies on Ganga’s microorganisms have primarily relied on cultivation-based methods, offering a restricted view of its bacterial diversity [12,13]. However, recent research utilizing advanced metagenomic approaches has begun to unveil a bacterial population capable of bioremediating significant contaminants in the river’s sediments [5,14,15,16]. A handful of studies have also employed metagenomic techniques to explore the presence of antibiotic resistance genes in the water and sediments of the Ganga [4,17,18].

The occurrence of microbial pollution presents additional risks, particularly due to the emergence of antibiotic-resistant bacteria (ARBs) and their associated ARGs [19]. The global public health community recognizes antibiotic resistance as a significant concern [20]. This issue is compounded by the likelihood of Horizontal Gene Transfer (HGT) events that facilitate the spread of ARGs among diverse microbial species [21]. In marine ecosystems, especially in coastal areas, antibiotic resistance is a prevalent phenomenon [19]. Human influences are major contributors to ARGs in environments impacted by anthropogenic activities [19]. Recent studies have linked the widespread occurrence of resistance genes primarily to fecal contamination [22]. Scientific investigations are increasingly focused on understanding the distribution, risks, and potential ecological consequences of antibiotics (ABs) and ARGs in various aquatic environments worldwide [20]. Additionally, there is an emerging hypothesis that resistance to heavy metals (HMs), evidenced through heavy metal resistance genes (HMRGs), may be linked to antibiotic (AB) resistance in environmental settings [23]. As a result, these resistance genes are crucial factors in assessing the ecological health of coastal waters.

Globally, various techniques have been established for assessing microbial pollution in aquatic environments, primarily focusing on detecting Fecal Indicator Bacteria (FIB) using cultivation-based methods. In contrast, high-throughput sequencing techniques, like shotgun metagenomics, offer a more advanced alternative to traditional microbial diversity studies. These innovative approaches facilitate a thorough investigation of environmental microbial communities. This includes examining the 16S rRNA genes to understand the breadth of microbial diversity and exploring genes related to pathogenicity, resistance to antibiotics and heavy metals, and virulence factors to evaluate their potential functions and impacts on ecosystems [24,25].

An analytical comparison of water and sediment provides a foundational understanding of the microbial interactions at the interface of these two environments. A recent study conducted a comparative analysis of the bacteriome and antibiotic resistance profile in the river Ganga, focusing on discerning the variations between samples taken from the river water and the sediment. This research undertakes a detailed comparison of both the bacteriome and ARGs within water and sediment samples collected across substantial stretches of the river Ganga.

## 2. Results

### 2.1. Sequencing Summary

The estimated sizes of the libraries varied between 8.4 Gb and 9.4 Gb (Table 1). The assembly statistics revealed that the average total contigs in the present study was 3,434,087. The average number of contigs ≥150 bp and ≤150 bp were found to be 3,307,063 and 127,024, respectively. The average total length of contigs was found to be 404,842,009 bp. A summary of all the statistics from all the samples is mentioned in Table 1.

### 2.2. Bacterial Diversity Analysis

**Figure 1:**
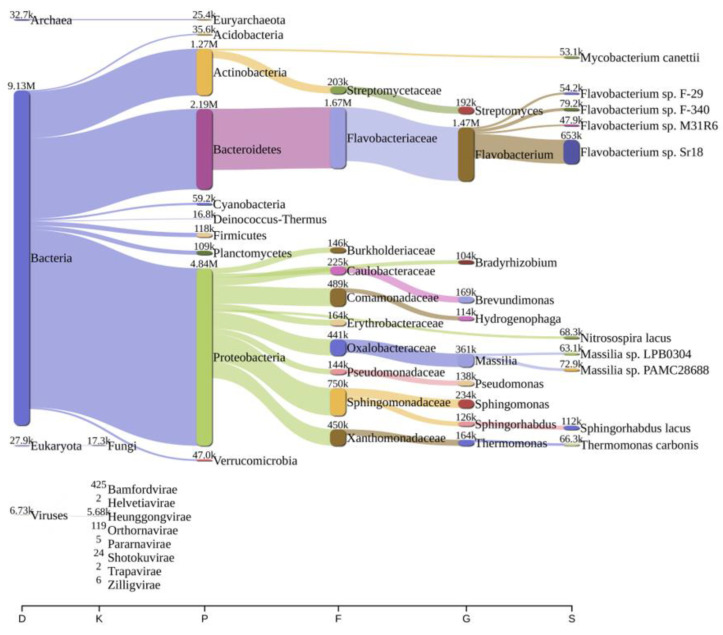
A sankey plot of comprehensive bacterial and viral community analysis in river Ganga sediment as analyzed from Pavian in Bageswar. This showcases the abundance and diversity of microbial taxa, including Proteobacteria, Flavobacterium, Sulfurospirillum, and various viral families. D: Domain, K: Kingdom, P: Phylum, F: Family, G: Genus, S: Species.

**Figure 2:**
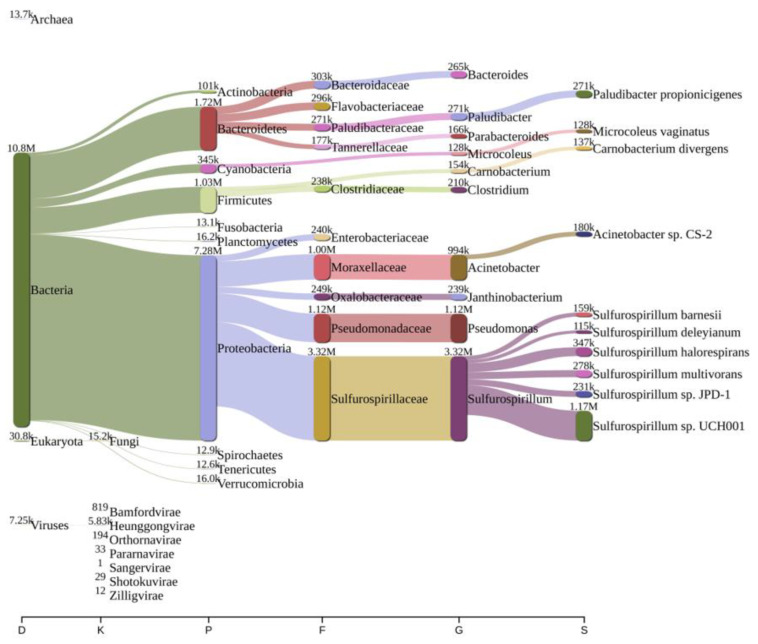
A sankey plot of comprehensive bacterial and viral community analysis in river Ganga sediment as analyzed from Pavian in Bagwan. This showcases the abundance and diversity of microbial taxa, including Proteobacteria, Flavobacterium, Sulfurospirillum, and various viral families. D: Domain, K: Kingdom, P: Phylum, F: Family, G: Genus, S: Species.

**Figure 3:**
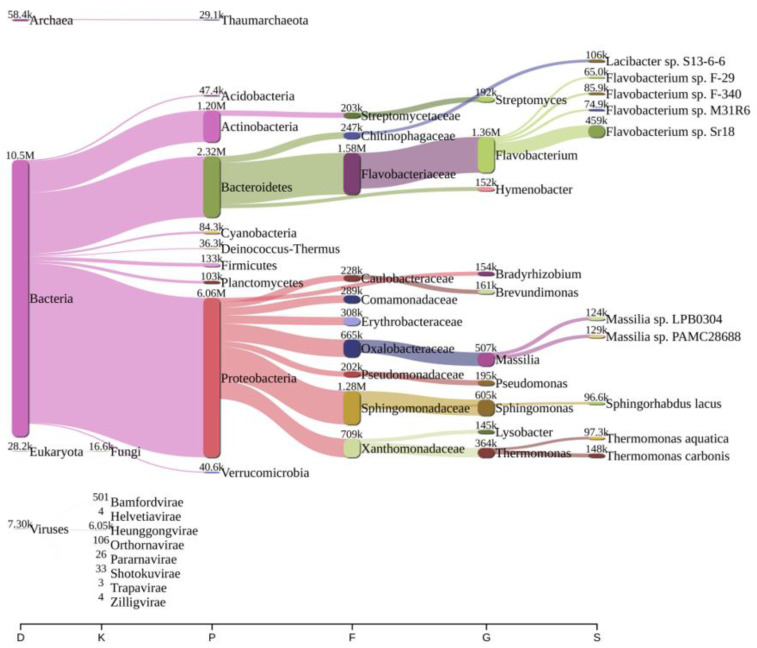
A sankey plot of comprehensive bacterial and viral community analysis in river Ganga sediment as analyzed from Pavian in Koteswar. This showcases the abundance and diversity of microbial taxa, including Proteobacteria, Flavobacterium, Sulfurospirillum, and various viral families. D: Domain, K: Kingdom, P: Phylum, F: Family, G: Genus, S: Species.

**Figure 4:**
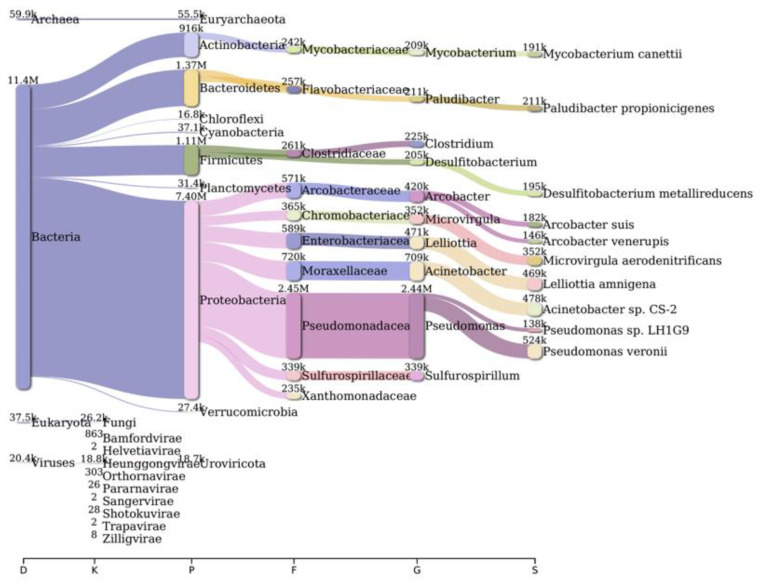
A sankey plot of comprehensive bacterial and viral community analysis in river Ganga sediment as analyzed from Pavian in Rasulabad Ghat. This showcases the abundance and diversity of microbial taxa, including Proteobacteria, Flavobacterium, Sulfurospirillum, and various viral families. D: Domain, K: Kingdom, P: Phylum, F: Family, G: Genus, S: Species.

**Figure 5:**
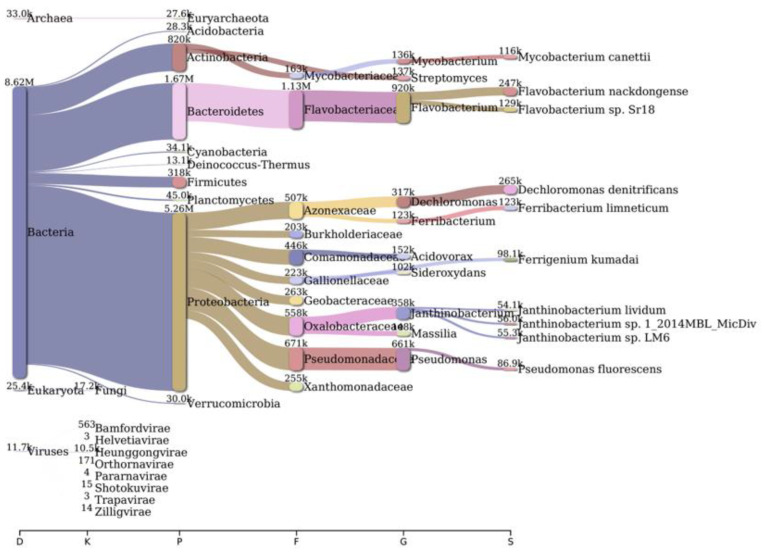
A sankey plot of comprehensive bacterial and viral community analysis in river Ganga sediment as analyzed from Pavian in Sahidabad. This showcases the abundance and diversity of microbial taxa, including Proteobacteria, Flavobacterium, Sulfurospirillum, and various viral families. D: Domain, K: Kingdom, P: Phylum, F: Family, G: Genus, S: Species.

**Figure 6:**
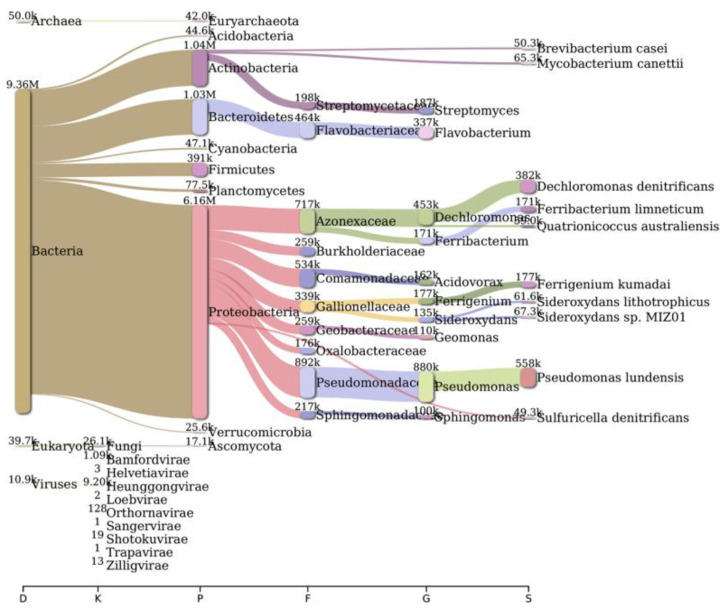
A sankey plot of comprehensive bacterial and viral community analysis in river Ganga sediment as analyzed from Pavian in Triveni Sangam. This showcases the abundance and diversity of microbial taxa, including Proteobacteria, Flavobacterium, Sulfurospirillum, and various viral families. D: Domain, K: Kingdom, P: Phylum, F: Family, G: Genus, S: Species.

Analysis of bacterial diversity conducted using Kraken2 and Pavian demonstrated that Proteobacteria were the most abundantly found across all surveyed locations in the river Ganga basin (Figure 1, Figure 2, Figure 3, Figure 4, Figure 5 and Figure 6). In the specific sites of Bageswar, Koteswar, and Sahidabad, there was a notable abundance of *Flavobacterium* spp. (Figure 1, Figure 3, and Figure 5, respectively). Meanwhile, in the Rasulabad Ghat and Triveni Sangam locations, Pseudomonas spp. were observed to be prevalent (Figure 4 and Figure 6, respectively). In Bagwan, *Sulfurospirillum* spp. ware dominant (Figure 2).

### 2.3. AMR Genes Abundance

Geographical variability in ARG profiles was studied in the present study. This investigation delineated the geographical distribution patterns of antimicrobial resistance (AMR) genes across five discrete sites: Bageswar, Bagwan, Rasulabad Ghat, Sahidabad, and Triveni Sangam (Table 2). We did not find any AMR in the Koteswar sample. We found diverse categories of AMR, including but not limited to Aminoglycoside, Streptomycin, and Cephalosporin. The outcomes provide critical insights into the regional dissemination and the prevalence of AMR determinants. Aminoglycoside resistance determinants such as *aac(6′)-Ib*, *aadS*, *acrD*, and *ANT(2″)-Ia* were predominantly detected in Rasulabad Ghat, with aadS also present in Bagwan and Triveni Sangam. Rasulabad Ghat exclusively exhibited the presence of Streptomycin resistance genes (*aadA1*, *aadA5*, *aadA6*). Beta-lactam resistance markers, particularly *blaOXA-209*, were observed in Bagwan and Rasulabad Ghat, while *blaOXA-119* was exclusively found in Triveni Sangam. The *acrB* gene, associated with multi-drug resistance, was identified in Bagwan and Rasulabad Ghat.

This study highlighted distinct regional disparities in the distribution of AMR genes. Triveni Sangam displayed the unique presence of *blaOXA-119*, which was absent in other studied areas. Bagwan and Rasulabad Ghat were characterized by a higher incidence of genes, such as *acrB*, *blaOXA-209*, and *baeR*, indicative of a broader spectrum of drug resistance. Conversely, Bageswar showed a minimal presence of the surveyed AMR genes. The detection of *blaRm3* in Bagwan and Sahidabad, a gene conferring resistance to a wide array of antibiotics, signals the emergence of high-level resistance in these regions. The exclusive identification of *blaTHIN-B* in Sahidabad, linked to carbapenem resistance, highlights specific regional challenges in antibiotic resistance.

### 2.4. Virulence Factor (VF) Abundance

Our comprehensive study examined the distribution of virulence genes and associated factors across multiple locations. Different contigs and their corresponding NCBI accession numbers, alongside the designation of virulence genes and factors, were identified in the present study and were exclusively linked to *Pseudomonas aeruginosa* (Table 3).

The analysis revealed the widespread distribution of flagella-associated genes (e.g., *flgC*, *flgG*, *flgH*, *flgI*) across various locations. The *flgC* (Flagella VF0273) was identified in Bagwan, Triveni Sangam, and Rasulabad Ghat only. The genes related to Type III and Type IV secretion systems, such as *pcrD*, *pscR*, *pcrH* in Triveni Sangam, and *pilG* in Triveni Sangam, Bagwan, and Sahidabad, were also identified. Alginate biosynthesis genes, like *algU* (Alginate VF0091) in Sahidabad and algI in Bagwan and Triveni Sangam, were detected. The presence of genes associated with pyochelin synthesis (*pchD*, *pchC*, *pchG*, *pchF*, *pchB*, *pchR*) exclusively in Rasulabad Ghat highlights the region-specific adaptation of bacteria in iron acquisition, a critical factor for bacterial survival and virulence. Moreover, *clpV1*, *hsiG1*, *dotU1*, *hcp1*, *hsiB1/vipA*, and *hsiC1/vipB* in Rasulabad Ghat, associated with a Type VI secretion system (HSI-I VF0334), were identified. This study also identified the *acpXL* gene in Bageswar, which was linked to LPS (CVF383) in *Brucella* sp.

### 2.5. KEGG Pathway Analysis

**Figure 7:**
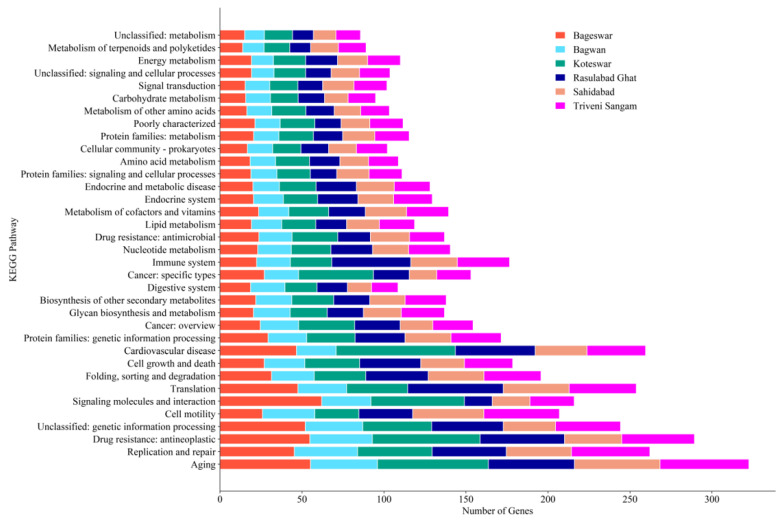
Graphical representation of the distribution of various biological processes in the Ganga River basin as analyzed from the KEGG. It depicts a comparative analysis across the six locations (Bageswar, Bagwan, Koteswar, Rasulabad Ghat, Sahidabad, and Triveni Sangam), showing the frequency of processes ranging from aging and replication to metabolism and drug resistance.

Our KEGG pathway analysis revealed a diverse range of biological processes across all six distinct locations in the river Ganga (Figure 7). Notably, antimicrobial resistance pathways were significantly represented, especially in Rasulabad Ghat and Sahidabad. Core metabolic pathways, including energy and carbohydrate metabolism, were uniformly present across all locations. The distribution of pathways related to diseases, such as cancer and endocrine disorders, varied among locations. There was a noticeable diversity in amino acid metabolism pathways, underscoring the metabolic adaptability of organisms in different environments. We also found unclassified pathways, possibly linked with genetic information processing and the metabolism process.

### 2.6. COG Analysis

**Figure 8:**
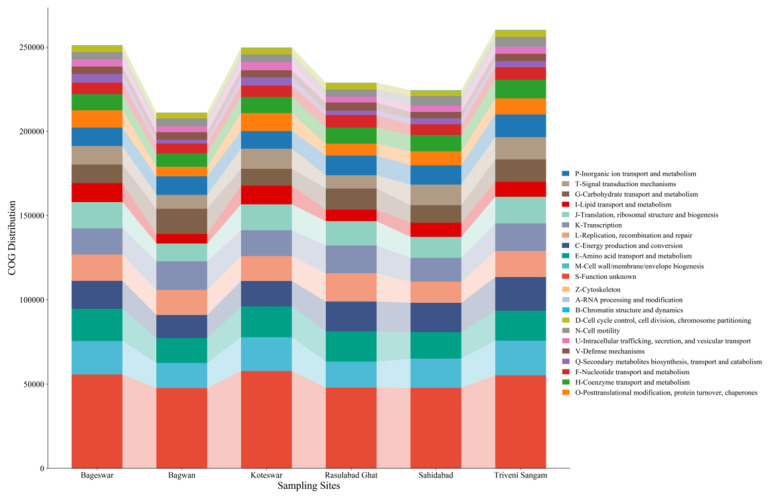
Bar chart displaying the Cluster of Orthologous Groups (COG) functional category distribution across the six locations in the Ganga River basin. The categories range from RNA processing and chromatin structure to metabolism and defense mechanisms, highlighting the diversity and abundance of microbial functions in these regions.

The Cluster of Orthologous Groups (COG) analysis conducted across the six locations revealed varied distributions of functional categories (Figure 8). The categories related to “Energy production and conversion”, “Amino acid transport and metabolism”, and “Carbohydrate transport and metabolism” were notably prevalent. Significant representation of “Cell cycle control, cell division, chromosome partitioning” and “Replication, recombination and repair” highlights the active cellular processes occurring in these environments. The variability in “Defense mechanisms”, “Signal transduction mechanisms”, and “Inorganic ion transport and metabolism” across locations were found. We also found a notable number of sequences under “Function unknown”.

## 3. Materials and Methods

### 3.1. Sample Collection

**Figure 9:**
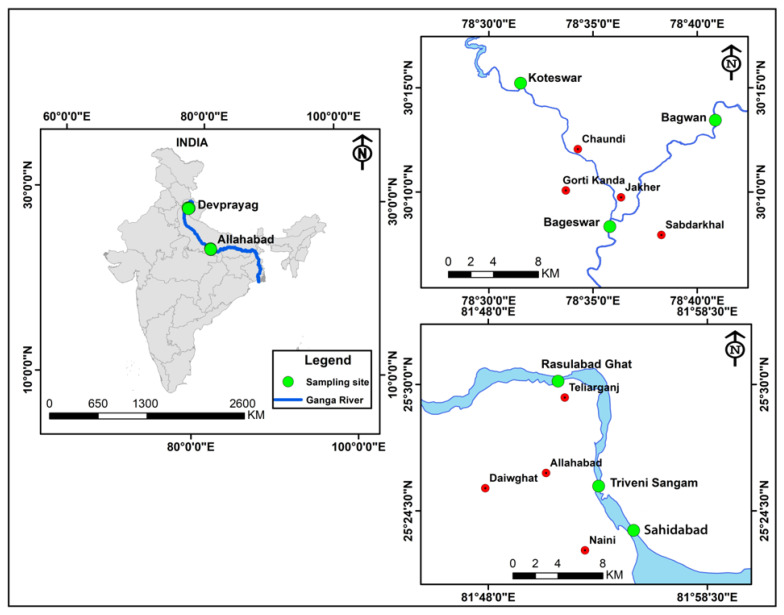
Map depicting the sampling sites along the Ganga River for the metagenomic study. Key locations include Bageswar, Bagwan, Koteswar, Rasulabad Ghat, Sahidabad, and Triveni Sangam spread across various geographical coordinates. Other locations near to the studied regions are marked with red circles.

Sediment and water samples were systematically collected from six distinct sites along the river Ganga. These locations included Koteswar (N 300.25′37.67″, E 780.52′54.77″), Bagwan (N 300.22′41.84″ E 780.68′12.99″), and Bageswar (N 300.13′90.18″ E 780.59′68.21″) near Devprayag, Uttarakhand, India, along with Rasulabad Ghat (N 250.50′24.64″ E 810.85′57.41″), Triveni Sangam (N 250.42′63.09″ E 810.88′81.93″), and Sahidabad (N 250.39′41.57″ E 810.91′61.04″) near Allahabad, Uttar Pradesh, India. Collections were conducted in the morning hours, between 8.30 and 10.30 AM, in March 2021 (Figure 9). From each location, approximately 500 g of sediment and 500 mL of water were collected. These samples were then meticulously stored in distinct autoclaved amber glass bottles (500 mL), clearly labeled as Koteswar, Bagwan, Bageswar, Rasulabad Ghat, Triveni Sangam, and Sahidabad. At every site, five sediment samples were obtained at intervals of approximately 200 m and subsequently combined to form a single representative sample for each location. The pH and temperature of each sample were measured on-site using an MT-222 Digiflexi digital thermometer (Dr. Morepen, New Delhi, India) and portable pH meter (Hanna Instrument, Sigma, St. Louis, MO, USA). All sediment samples were carefully placed in sterile plastic bags, securely sealed, and transported on ice (4 °C), and subsequently stored at −20 °C for further analysis.

### 3.2. Genomic DNA Isolation, Library Preparation, and Sequencing

Genomic DNA was extracted from the collected sediment samples using the XpressDNA Soil Kit (MagGenome, Union City, CA, USA), with certain modifications to the standard protocol. The integrity and concentration of the extracted DNA were assessed using 1% agarose gel electrophoresis and Nanodrop™ (Thermo Scientific, Waltham, MA, USA), respectively, and the samples were subsequently preserved at −20 °C for future analysis. A criterion for the DNA library construction was established, requiring an optical density (OD) absorbance between 1.8 and 2.0 at a 260/280 nm purity ratio and a minimum DNA concentration of 1 μg.

**Figure 10:**
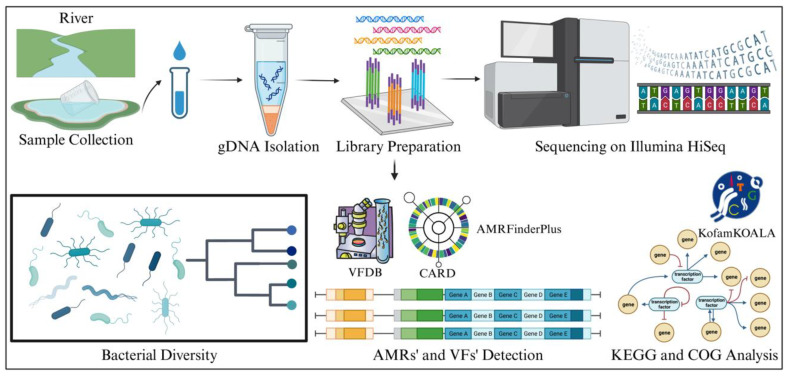
Illustration of the methodological framework of the present study, starting from sample collection through sequencing and analysis. It includes following methodologies like genomic DNA (gDNA) isolation, library preparation, and Illumina HiSeq sequencing. The computational workflow of bacterial diversity assessment, the identification of virulence factors (VFs) from the VFDB, and the analysis of antibiotic resistance using CARD and AMRFinderPlus are shown. The final stages involve the detection of AMR and VFs, followed by KEGG and COG analysis for comprehensive genetic evaluation.

The purified DNA was sent to Genotypic Technology Pvt. Ltd. (Bangalore, India) for library preparation and sequencing. Briefly, a NEBNext Ultra DNA Library Prep Kit (Ipswich, MA, USA) was employed to prepare the paired-end sequencing library, following the manufacturer’s protocol. The DNA fragments were then purified using a MinElute PCR Purification Kit (Qiagen, Ltd., Crawley, UK). Post-preparation, the libraries were subjected to DNA segmentation quantification in conjunction with HyperLadder IV (Bioline, London, UK) to ascertain the size of the DNA library. In line with Illumina’s standard protocol, the libraries were pooled at equal molar concentrations for sequencing. An Illumina HiSeq 2500 (San Diego, CA, USA) quick run of 2 × 150 bp was utilized for sequencing, and duplicate samples were allocated over two lanes for comprehensive sequencing. The detailed workflow used in this study is shown in Figure 10.

### 3.3. Bacterial Diversity Detection

Taxonomic profiling of six metagenomic samples was conducted utilizing the NCBI taxonomy dataset. For each sample, a taxonomic tree was constructed by employing the neighbor-joining method facilitated by MEGAN6 [26] and Kraken2 v2.1.3 [27]. The Kraken2 report file was finally used for generating a bacterial diversity classification plot or Sankey plot using Pavian v1.0.

### 3.4. Functional Analysis

In the present river sediment metagenomic sample analysis, the assembly file was annotated using PROKKA v1.14.5 [28]. The annotated sequence was used in the Virulence Factors Database (VFDB) to determine virulence factors. To ascertain the presence of ARGs within the river sediment, various databases, including the Comprehensive Antibiotic Resistance Database (CARD), NCBI, and Resfinder, were employed. For the annotation of core orthologues, consensus sequences were subjected to BLAST analysis against KOfam, a database of KEGG orthologues, employing kofamKOALA [29]. Following this, the eggNOG-mapper tool [30], in conjunction with the EggNOG database [31], was utilized to systematically categorize all core orthologue sequences into clusters of orthologous groups of proteins (COGs).

## 4. Discussion

The observed bacterial diversity and the prevalence of specific taxa, in particular locations of the river Ganga basin, emphasize the intricate relationship between microbial communities and their environmental conditions. These findings have significant implications for understanding the ecological health and biogeochemical processes within the river system.

The predominance of Proteobacteria across all examined locations aligns with the existing literature, which often cites Proteobacteria as a dominant phylum in aquatic environments [32,33,34]. This ubiquity can be attributed to the diverse metabolic capabilities of Proteobacteria, allowing them to thrive in various environmental conditions. In specific sites, like Bageswar, Koteswar, and Sahidabad, the marked prevalence of *Flavobacterium* spp. is noteworthy. *Flavobacterium* is known for its role in nutrient cycling and has been previously identified in freshwater ecosystems [35]. Its abundance in these areas might indicate specific ecological functions, possibly related to the organic matter degradation or nitrogen cycle in these river segments. Furthermore, the distinct presence of *Sulfurospirillum* spp. in Bagwan and Triveni Sangam and *Pseudomonas* spp. in Sahidabad deserves attention. *Sulfurospirillum* spp. are known for their role in sulfur cycling and have been identified in environments with low oxygen levels [36], which might suggest specific anoxic conditions or sulfur-rich environments in these parts of the river Ganga. On the other hand, *Pseudomonas* spp., known for its metabolic versatility and adaptability, might indicate a high level of organic pollutants or anthropogenic influence in Sahidabad, as these bacteria are often associated with contaminated sites.

Our investigation into the distribution of antimicrobial resistance (AMR) genes across multiple geographical locations revealed a complex and diverse landscape of resistance mechanisms. This study encompassed a wide array of resistance types, including Aminoglycoside, Streptomycin, Cephalosporin, Penam, and others, across six locations in river Ganga, i.e., Bageswar, Bagwan, Koteswar, Rasulabad Ghat, Sahidabad, and Triveni Sangam.

The findings demonstrated significant variability in the presence and prevalence of specific AMR genes among the studied locations. For example, genes conferring resistance to Aminoglycosides like *aac(6’)-Ib*, *aadS*, *acrD*, and *ANT(2″)-Ia* were predominantly identified in Rasulabad Ghat. This suggests a localized emergence or higher usage of aminoglycoside antibiotics in this area, leading to selective pressure and subsequent development of resistance. The role of selective pressure on antibiotic resistance has been well reviewed in an earlier work [37] and well studied in *P. aeroginosa* [38]. The detection of *aadS* in Bagwan and Triveni Sangam further indicates the spread of this resistance mechanism beyond a single locality. Notably, Streptomycin resistance genes such as *aadA1*, *aadA5*, and *aadA6* showed a similar pattern of being exclusively found in Rasulabad Ghat. This further supports the hypothesis of region-specific antibiotic usage or resistance development mechanisms. The region-specific antimicrobial resistance has been described in earlier studies on *Streptococcus pneumoniae* [39], *Mycobacterium tuberculosis* [40], and *Klebsiella pneumoniae* [41]. Beta-lactam resistance genes, like *blaOXA-209,* which are unique to Bagwan and Rasulabad Ghat, and *blaOXA-119* in Triveni Sangam, underscore the heterogeneity in the distribution of resistance genes. These genes have been well studied in an earlier work on the members of genus *Tenacibaculum* [42].

Furthermore, the gene *acrB*, associated with resistance to a broad spectrum of antibiotics, was observed in Bagwan and Rasulabad Ghat, which shows the presence of multi-drug resistant strains in these areas. The *acrB* gene encodes a heterotrimeric protein that forms a component of the inner membrane and is primarily tasked with substrate recognition and energy transduction. It functions as a drug/proton antiporter, playing a pivotal role in these processes [43,44]. The occurrence of blaRm3, a gene showing resistance to a wide range of antibiotics, in Bagwan and Sahidabad, and *blaTHIN-B* in Sahidabad, points toward the emergence of high-level antibiotic resistance in these areas. This gene is one of the key genes related to antibiotic resistance and has been well studied as an indicator of antibiotic resistance in various water sources [45,46,47]. In addition, the minimal presence of the surveyed AMR genes in Bageswar indicates a possible lower prevalence of resistant strains or divergent antibiotic utilization patterns in this locality. A study examining AMR genes in the Ili River reported a lower occurrence of these genes, indicating minimal human intervention in certain areas [48]. So, the AMR genes analyzed in the present study would help in finding the effect of human intervention on the upper and lower river Ganga basin.

The study also revealed that the distribution of these resistance genes is not uniform across the regions, indicating a complex interplay of factors such as local antibiotic usage patterns, environmental conditions, and genetic exchange mechanisms that might contribute to this varied distribution. The distribution and diversity of AMR genes across the studied locations provide a crucial understanding of the regional dynamics of antibiotic resistance. The findings highlight the necessity for targeted surveillance and stewardship programs to monitor and manage the spread of AMR in these specific areas. Understanding the patterns of resistance gene prevalence can aid in developing strategic interventions to curb the burgeoning issue of antibiotic resistance in diverse geographical settings.

The current investigation into the distribution of virulence factors across various locations offers significant insights into the adaptive mechanisms of pathogenic bacteria, particularly *Pseudomonas aeruginosa*. This study underscores the complexity of bacterial virulence and its dependency on environmental context.

The detection of flagella-associated genes such as *flgC*, *flgG*, *flgH*, and *flgI* in multiple locations implies a widespread reliance on motility and adherence as critical virulence factors. The prevalence of these genes across diverse geographical areas signifies a common strategy employed by bacteria to establish infection and colonization. This consistency in virulence gene distribution suggests a potential universal response to similar environmental pressures or host interactions [49].

The identification of genes related to Type III and Type IV secretion systems in specific locations, like Triveni Sangam and Sahidabad, indicates the presence of advanced bacterial systems for effector protein delivery. These secretion systems are pivotal in bacterial pathogenesis, facilitating direct interactions with host cells [50,51]. The localized presence of these genes may reflect regional variations in bacterial–host dynamics or environmental factors that favor certain pathogenic strategies. Alginate biosynthesis genes, particularly *algU* and *algI*, highlight the capability of bacterial populations in these regions to form biofilms. Biofilms confer significant advantages to bacteria, including enhanced antibiotic resistance and protection from host immune responses [52]. The regional distribution of these genes suggests environmental or selective pressures favoring biofilm-forming strains, potentially due to their survival and persistence advantages in specific niches. The role of biofilm in bacterial survival in the river Ganga basin has been studied in an earlier work [53,54]. The exclusive presence of pyochelin synthesis genes in Rasulabad Ghat points to an environment where iron acquisition is a crucial survival factor. Iron is a vital nutrient for bacterial growth, and its acquisition is often a limiting factor in pathogenic success. The specificity of these genes to Rasulabad Ghat may indicate unique iron availability or competition dynamics in this site.

The presence of Type VI secretion system genes in Rasulabad Ghat suggests an environment rich in bacterial competition. This system is known for its role in bacterial warfare, allowing for the delivery of toxins into competing bacterial cells [55,56]. The concentration of these genes in one location might reflect a high-density bacterial community with intense inter-bacterial interactions.

The geographical variability in virulence gene profiles poses challenges for infection control and management strategies. Understanding the specific virulence factors in Ganga River water can aid in developing targeted therapeutic and preventive measures. This study highlights the need for the localized surveillance of pathogenic bacteria to better understand and combat region-specific infectious challenges.

The distinct distribution of genes in processes like aging, replication, repair, and cell motility across studied locations underscore the unique ecological characteristics of each site. This diversity can be attributed to a multitude of factors, including environmental conditions, the presence of specific microbial communities, and local selective pressures. The variability observed in the data reflects the adaptive responses of organisms to their respective habitats. The adaptive response of microbial communities to the environment has been studied in various research works, reflecting its importance in future drug design [57,58].

The genes related to antimicrobial resistance, particularly in regions like Rasulabad Ghat and Sahidabad, raise significant public health concerns. This observation suggests that these areas might be reservoirs of drug-resistant organisms, potentially due to the overuse of antibiotics or the presence of other selective agents. The data necessitate a more focused approach toward monitoring and managing antimicrobial resistance in these regions. The uniform distribution of primary metabolic activities, such as energy and carbohydrate metabolism across all locations, indicates the fundamental nature of these processes in sustaining life. However, the variation in amino acid metabolism across different regions highlights the metabolic flexibility and adaptability of the residing organisms, allowing them to thrive in diverse environmental conditions. The presence of disease-related categories, like cancer and endocrine disorders, in the data suggests potential environmental or genetic factors influencing disease prevalence in these regions. These findings could be instrumental in guiding further epidemiological studies to explore the environmental contributions to disease etiology.

A significant portion of the genes fell into unclassified categories, pointing to the existence of unknown or poorly understood biological processes in these regions. This observation opens avenues for future research aimed at uncovering novel biological functions and mechanisms, which could have far-reaching implications on understanding ecosystem dynamics and organismal adaptations.

The Cluster of Orthologous Groups (COG) analysis, encompassing six distinct geographical locations, unveiled a rich tapestry of functional biodiversity. This diversity, evident in the distribution of various COG categories, reflects the intricate interplay between microbial communities and their respective environments.

Central to our findings is the representation of categories related to energy production, carbohydrate metabolism, and amino acid transport across all studied regions. This uniformity in metabolic profiles suggests a fundamental role of these processes in sustaining microbial life [59,60,61]. It underscores the universality of certain metabolic functions, which serve as the cornerstone for microbial survival and proliferation, irrespective of geographical variances.

Further study revealed the genes involved in cellular processes, particularly cell cycle control, cell division, and chromosomal dynamics. The marked presence of these categories indicates active cellular mechanisms, potentially as a response to local environmental pressures or genomic instabilities. This observation aligns with the notion that microorganisms are in a constant state of adaptation, modifying their cellular processes to optimize survival and efficiency in diverse habitats, which supports various studies related to this [62,63,64].

The variation observed in defense mechanisms and signal transduction pathways among the different locations indicates the adaptive strategies employed by microbial communities. This variability could stem from the need to respond to specific local environmental conditions, such as nutrient availability, the presence of antimicrobial agents, or other ecological pressures [65]. The differential expression of these categories highlights the role of local environmental factors in shaping the functional capabilities of microbial communities.

A particularly intriguing aspect of our analysis is the substantial proportion of sequences classified under “Function unknown”. This finding points to a significant gap in our understanding of microbial functional diversity and suggests the presence of novel or poorly understood biological processes within these communities. It opens avenues for future research to explore these uncharacterized functions, which could lead to groundbreaking discoveries in microbial ecology and biology.

## 5. Conclusions

In conclusion, this study offers critical insights into the bacterial diversity across six regions of the river Ganga through a metagenomics approach. In addition to this, the spatial distribution of AMR and virulence factors, illustrating a complex and diverse landscape of antibiotic resistance across various geographical locations on the river Ganga, has been depicted. The findings emphasize the importance of region-specific public health strategies and the need to integrate these with local environmental and socioeconomic contexts to effectively combat AMR. This study also highlights the intricate relationship between organisms and their environments, as evidenced by the diversity of biological processes observed in different locations. This emphasizes the need for local environmental considerations in both ecological and biological research and for region-specific strategies to address public health challenges, such as AMR. The research contributes significantly to our understanding of the geographical distribution of bacterial virulence factors and the importance of environmental and regional factors in bacterial pathogenesis. While the study provides valuable insights, it is limited by its focus on specific resistance genes and geographical locations. Future research should broaden to include a wider array of resistance determinants and environmental samples to fully comprehend AMR dynamics. This expansion is crucial for the global fight against the growing threat of antimicrobial resistance and for developing effective infection control and management strategies. Understanding the diverse functional profiles of microbial communities and their adaptability will be crucial to addressing ecological dynamics and ensuring environmental sustainability.

## Figures and Tables

**Table 1:** Summary of statistics from metagenome assembly.

Parameters	Bageswar	Bagwan	Koteswar	Rasulabad Ghat	Sahidabad	Triveni Sangam
Contigs (≤150 bp)	460,537	71,161	87,682	40,754	43,771	58,237
Contigs (≥150 bp)	1,008,926	1,766,259	5,172,327	3,263,830	3,839,878	4,791,158
Total contigs	1,469,463	1,837,420	5,260,009	3,304,584	3,883,649	4,849,395
Largest contig	887,047	897,272	1,192,971	343,376	789,499	645,470
Total length (in bp)	468,274,061	340,847,742	444,765,440	366,965,171	374,775,947	433,423,693
GC content (in %)	52	40	52	46	50	47
N50	1091	2615	1237	1517	1402	1471
N90	558	619	561	578	573	575
L50	91,382	21,741	66,190	41,780	48,856	55,263
L90	346,262	148,883	298,653	215,191	232,472	263,312
Ns per 100 kbp	0	0	0	0	0	0

**Table 2:** List of AMRs found in the present study across the samples. “F” and “NF” represent whether the AMR was found or not found, respectively, across the samples.

Resistance	Gene Name	Number of Reads				
		Bageswar	Bagwan	Rasulabad Ghat	Sahidabad	Triveni Sangam
Aminoglycoside	*aac(6′)-Ib*	NF	NF	F	NF	NF
Streptomycin	*aadA1*	NF	NF	F	NF	NF
Streptomycin	*aadA5*	NF	NF	F	NF	NF
Streptomycin	*aadA6*	NF	NF	F	NF	NF
Aminoglycoside	*aadS*	NF	F	F	NF	F
Cephalosporin; Fluoroquinolone; Glycylcycline; Penam; Phenicol; Rifamycin; Tetracycline; Triclosan	*acrB*	NF	F	F	NF	NF
Aminoglycoside	*acrD*	NF	F	NF	NF	NF
Penam	*AER-1*	NF	NF	F	NF	NF
Aminoglycoside	*ANT(2″)-Ia*	NF	NF	F	NF	NF
Aminoglycoside	*ANT(3″)-Ia*	NF	NF	F	NF	NF
Aminoglycoside	*ANT(6)-Ia*	NF	NF	F	NF	NF
Aminoglycoside	*aph(3″)-Ib*	NF	NF	F	NF	F
Aminoglycoside	*aph(6)-Id*	NF	NF	F	NF	NF
Peptide	*arnA*	NF	F	NF	NF	NF
Rifamycin	*arr-2*	NF	NF	F	NF	NF
Peptide	*bacA*	NF	F	F	NF	NF
Aminocoumarin; Aminoglycoside	*baeR*	NF	F	F	NF	NF
BETA-LACTAM	*bla-A*	NF	NF	F	NF	NF
BETA-LACTAM	*blaAER-1*	NF	NF	F	NF	NF
Carbapenem	*blaGES-14*	NF	NF	F	NF	NF
Carbapenem	*blaGES-5*	NF	NF	F	NF	NF
BETA-LACTAM	*blaMCA*	NF	NF	F	NF	NF
BETA-LACTAM	*blaOXA-119*	NF	NF	NF	NF	F
BETA-LACTAM	*blaOXA-209*	F	NF	F	NF	NF
BETA-LACTAM	*blaOXA-296*	NF	F	NF	NF	NF
BETA-LACTAM	*blaOXA-347*	NF	NF	F	NF	NF
Carbapenem; Cephalosporin; Penam	*blaRm3*	NF	F	NF	F	NF
BETA-LACTAM	*blaRSD1-1*	NF	NF	NF	NF	F
Carbapenem	*blaTHIN-B*	NF	NF	NF	F	NF
Cephalosporin	*blaVEB-9*	NF	NF	F	NF	NF
Phenicol	*catQ*	NF	F	F	NF	NF
Aminoglycoside; Fluoroquinolone	*ceoB*	NF	NF	NF	F	F
Phenicol	*cmlA5*	NF	NF	F	NF	NF
Aminocoumarin; Aminoglycoside	*cpxA*	NF	NF	F	NF	NF
Fluoroquinolone; Macrolide; Penam	*CRP*	NF	F	F	NF	NF
Trimethoprim	*dfrA3*	NF	F	NF	NF	NF
Trimethoprim	*dfrG*	NF	NF	F	NF	NF
Fluoroquinolone	*emrR*	NF	F	F	NF	NF
Cephalosporin; Fluoroquinolone; Glycylcycline; Penam; Phenicol; Rifamycin; Tetracycline; Triclosan	*Enterobacter cloacae* *acrA*	NF	F	F	NF	NF
Macrolide	*ere(D)*	NF	NF	F	NF	NF
Chloramphenicol	*EstDL136*	F	NF	NF	NF	NF
Fosfomycin	*fos1*	NF	F	NF	NF	NF
Cephalosporin; Cephamycin; Fluoroquinolone; Macrolide; Penam; Tetracycline	*H-NS*	NF	F	F	NF	NF
Aminoglycoside; Carbapenem; Cephalosporin; Fluoroquinolone; Macrolide; Penam; Peptide	*Klebsiella pneumoniae* *KpnH*	NF	F	F	NF	NF
Aminoglycoside; Carbapenem; Cephalosporin; Fluoroquinolone; Macrolide; Penem; Peptide	*KpnG*	NF	NF	F	NF	NF
Lincosamide	*lnu(D)*	NF	NF	F	NF	NF
Carbapenem; Cephalosporin; Cephamycin; Fluoroquinolone; Glycylcycline; Monobactam; Penem;phenicol; Rifamycin; Tetracycline; Triclosan	*marA*	NF	F	F	NF	NF
Aminocoumarin	*mdtB*	NF	F	F	NF	NF
Aminocoumarin	*mdtC*	NF	F	F	NF	NF
Macrolide	*mefA*	NF	NF	F	NF	NF
Macrolide	*mefB*	NF	NF	F	NF	NF
Macrolide	*mefC*	NF	NF	F	NF	NF
Lincosamide; Macrolide; Oxazolidinone; Phenicol; Pleuromutilin; Streptogramin; Tetracycline	*mel*	NF	NF	F	NF	NF
Aminocoumarin; Aminoglycoside; Cephalosporin; diaminopyrimidine; Fluoroquinolone; Macrolide; penam; Phenicol; Tetracycline	*MexD*	NF	NF	F	NF	NF
Diaminopyrimidine; Fluoroquinolone; Phenicol	*MexF*	NF	F	F	NF	F
Macrolide	*mphE*	NF	F	F	NF	NF
Macrolide	*mphF*	NF	NF	F	NF	NF
Nitroimidazole	*msbA*	NF	F	F	NF	NF
Erythromycin; Azithromycin; Telithromycin; Quinupristin; Pristinamycin_IA; Virginiamycin_S	*msr(D)*	NF	NF	F	NF	NF
Macrolide	*msr(E)*	F	F	F	NF	NF
Diaminopyrimidine; Fluoroquinolone; Glycylcycline; Nitrofuran; Tetracycline	*oqxA*	NF	NF	F	NF	NF
Diaminopyrimidine; Fluoroquinolone; Glycylcycline; Nitrofuran; Tetracycline	*oqxB*	NF	NF	F	NF	NF
Aminocoumarin; Aminoglycoside; Carbapenem;cephalosporin; Cephamycin; Diaminopyrimidine; Fluoroquinolone; Macrolide; Monobactam; Penem; Peptide; Phenicol; Sulfonamide; Tetracycline	*Pseudomonas aeruginosa* *CpxR*	NF	NF	F	NF	NF
Fluoroquinolone	*qnrD2*	NF	F	NF	NF	NF
Carbapenem; Cephalosporin; Cephamycin; Fluoroquinolone; Glycylcycline; Monobactam; Penam; Phenicol; Rifamycin; Tetracycline; Triclosan	*ramA*	NF	F	F	NF	NF
Rifamycin	*rphB*	NF	NF	F	NF	NF
Aminoglycoside	*spw*	NF	NF	F	NF	NF
Sulfonamide	*sul1*	NF	NF	F	NF	F
Sulfonamide	*sul2*	NF	NF	F	NF	F
Sulfonamide	*sul4*	NF	NF	F	F	NF
Tetracycline	*tet(36)*	NF	F	F	NF	NF
Tetracycline	*tet(39)*	F	F	F	NF	NF
Tetracycline	*tet(A)*	NF	NF	F	NF	NF
Tetracycline	*tet(G)*	NF	NF	F	NF	NF
Doxycycline; Tetracycline; Minocycline	*tet(M)*	NF	NF	F	NF	NF
Doxycycline; Tetracycline; Minocycline	*tet(O)*	NF	NF	F	NF	NF
Doxycycline; Tetracycline; Minocycline	*tet(Q)*	NF	NF	F	NF	NF
Doxycycline; Tetracycline; Minocycline	*tet(X)*	NF	NF	F	NF	NF
Tetracycline	*tetC*	NF	NF	F	NF	NF
Aminocoumarin; Aminoglycoside; Carbapenem; Cephalosporin; Cephamycin; Fluoroquinolone; Glycylcycline; Macrolide; Penam; Peptide; Phenicol; Rifamycin; Tetracycline; Triclosan	*tolC*	NF	F	NF	NF	NF

**Table 3:** List of virulence factors found across sediment samples in the river Ganga. The virulence factors have been categorized across samples and virulence factors.

Contig Id	Location	NCBI Accession No.	Virulence Gene	Virulence Factor
contigs_2469; contigs_1; contigs_240	Bagwan; Triveni Sangam; Rasulabad Ghat	NP_249769	*flgC*	Flagella (VF0273) (*Pseudomonas aeruginosa*)
contigs_45; contigs_11691; contigs_19; contigs_3945	Rasulabad Ghat; sahidabad; Triveni Sangam; Bagwan	NP_249773	*flgG*	
contigs_45	Rasulabad Ghat	NP_250143	*flhA*	
contigs_45; contigs_641; contigs_19	Rasulabad Ghat; Bagwan; Triveni Sangam	NP_250137	*fliP*	
contigs_45	Rasulabad Ghat	NP_250138	*fliQ*	
contigs_11691; contigs_19; contigs_3945	Sahidabad; Triveni Sangam; Bagwan	NP_249774	*flgH*	
contigs_45; contigs_641; contig_19; contigs_366	Rasulabad Ghat; Bagwan; Triveni Sangam; Bagwan	NP_250134	*fliM*	
contigs_45; contigs_641; contig_19; contigs_366	Rasulabad Ghat; Bagwan; Triveni Sangam; Bagwan	NP_249793	*fliG*	
contigs_45; contigs_11691; contigs_19; contigs_3945	Rasulabad Ghat; sahidabad; Triveni Sangam; Bagwan	NP_249775	*flgI*	
contigs_45; contigs_641; contig_19; contigs_366	Rasulabad Ghat; Bagwan; Triveni Sangam; Bagwan	NP_250145	*fleN*	
contigs_45; contigs_641; contigs_19	Rasulabad Ghat; Bagwan; Triveni Sangam	NP_249795	*fliI*	
contigs_45; contigs_641	Rasulabad Ghat; Bagwan	NP_249788	*fleQ*	
contigs_4	Triveni Sangam	NP_250394	*pcrD*	Type III TTSS (VF0083) (*Pseudomonas aeruginosa*)
contigs_4	Triveni Sangam	NP_250384	*pscR*	
contigs_4	Triveni Sangam	NP_250398	*pcrH*	
contigs_68665	Sahidabad	NP_249453	*algU*	Alginate (VF0091) (*Pseudomonas aeruginosa*)
contigs_5362; contigs_1	Bagwan; Triveni Sangam	NP_252238	*algI*	
contigs_1	Triveni Sangam	NP_252231	*alg8*	
contigs_42942	Rasulabad Ghat	NP_273273	*katA*	
contigs_41	Rasulabad Ghat	NP_460110	*csgG*	
contigs_45	Rasulabad Ghat	NP_251103	*pvdH*	
contigs_45; contigs_53	Rasulabad Ghat; Triveni Sangam	NP_251116	*pvdS*	
contigs_665	Rasulabad Ghat	NP_252911	*fptA*	
contigs_76423	Bagwan	BAA94855	*astA*	
contigs_2	Rasulabad Ghat	NP_253699	*waaF*	
contigs_45; contigs_269352	Rasulabad Ghat; Sahidabad	NP_251102	*mbtH-like*	
contigs_627	Bagwan	AAF37887	*ompA*	
contigs_5362	Bagwan	NP_252241	*algA*	
contigs_665	Rasulabad Ghat	NP_252918	*pchD*	Pyochelin (VF0095) (*Pseudomonas aeruginosa*)
contigs_665	Rasulabad Ghat	NP_252919	*pchC*	
contigs_665	Rasulabad Ghat	NP_252914	*pchG*	
contigs_665	Rasulabad Ghat	NP_252915	*pchF*	
contigs_665	Rasulabad Ghat	NP_252920	*pchB*	
contigs_665	Rasulabad Ghat	NP_252917	*pchR*	
contigs_4; contigs_24665; contigs_67305; contigs_39	Triveni Sangam; Bagwan; sahidabad; Bagwan	NP_249099	*pilG*	Type IV pili (VF0082) (*Pseudomonas aeruginosa*)
contigs_24665; contigs_67305; contigs_39	Bagwan; Sahidabad; Bagwan	NP_249100	*pilH*	
contigs_39	Bagwan	NP_249086	*pilT*	
contigs_622	Rasulabad Ghat	NP_248780	*clpV1*	Type VI HSI-I (VF0334) (*Pseudomonas aeruginosa*)
contigs_622	Rasulabad Ghat	NP_248778	*hsiG1*	
contigs_622	Rasulabad Ghat	NP_248768	*dotU1*	
contigs_622	Rasulabad Ghat	NP_248775	*hcp1*	
contigs_622	Rasulabad Ghat	NP_248773	*hsiB1/vipA*	
contigs_622	Rasulabad Ghat	NP_248774	*hsiC1/vipB*	
contigs_239842	Bageswar	NP_540392	*acpXL*	LPS (CVF383) (Brucella)
contigs_240	Rasulabad Ghat	NP_249768	*flgB*	Deoxyhexose linking sugar 209 Da capping structure (AI138) (*Pseudomonas aeruginosa*)
contigs_641	Bagwan	NP_250146	*fliA*	
contigs_204	Rasulabad Ghat	NP_254009	*algC*	Alginate biosynthesis (CVF522) (*Pseudomonas aeruginosa*)

## Data Availability

Metagenome data derived from our study were deposited to the NCBI Sequence Read Archive (SRA), which can be accessed via SRR16085958 (Koteswar), SRR16085957 (Bagwan), SRR16085956 (Bageswar), SRR16085955 (Rasulabad Ghat), SRR16085954 (Triveni Sangam), and SRR16085953 (Sahidabad).
